# Etalon Array Reconstructive Spectrometry

**DOI:** 10.1038/srep40693

**Published:** 2017-01-11

**Authors:** Eric Huang, Qian Ma, Zhaowei Liu

**Affiliations:** 1 Department of Physics, University of California, San Diego, 9500 Gilman Drive, La Jolla, California 92093-0407, USA; 2 Department of Electrical and Computer Engineering, University of California, San Diego, 9500 Gilman Drive, La Jolla, California 92093-0407, USA

## Abstract

Compact spectrometers are crucial in areas where size and weight may need to be minimized. These types of spectrometers often contain no moving parts, which makes for an instrument that can be highly durable. With the recent proliferation in low-cost and high-resolution cameras, camera-based spectrometry methods have the potential to make portable spectrometers small, ubiquitous, and cheap. Here, we demonstrate a novel method for compact spectrometry that uses an array of etalons to perform spectral encoding, and uses a reconstruction algorithm to recover the incident spectrum. This spectrometer has the unique capability for both high resolution and a large working bandwidth without sacrificing sensitivity, and we anticipate that its simplicity makes it an excellent candidate whenever a compact, robust, and flexible spectrometry solution is needed.

Spectrometry is used in nearly every field of scientific research, and as with all instruments, there are continuing efforts to make spectrometers cheaper and more compact. Nevertheless, developments in fabrication techniques and integrated electronics has allowed for significant advances in small, cheap spectrometers^1^,^2^,^3^,^4^,^5^,^6^,^7^,^8^. In particular, there is a large interest in filter-array based spectrometry methods^9^,^10^,^11^,^12^, where a 2D array of filters is integrated directly onto an image sensor, typically a CCD or CMOS camera sensor. These types of spectrometers can be very small and have no moving parts, leading to a robust and portable form factor. However, advances in signal processing techniques have now allowed for more efficient ways to acquire a signal through algorithmic reconstruction.

## Theory

**Figure 1:**
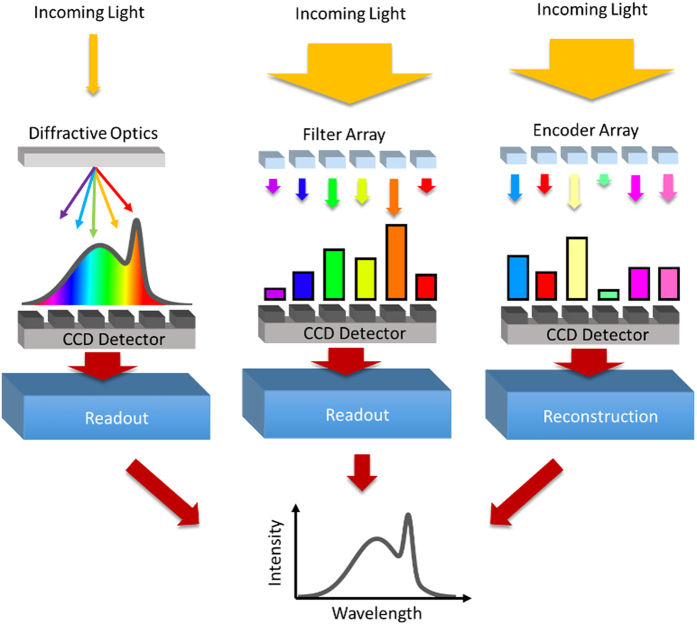
In conventional spectrometry (left), a diffraction grating is used to spatially separate the incoming light by color, and the intensity of each color is then read by a linear light detector array. In a filter array spectrometer (center), an array of bandpass filters each transmit a small wavelength range, which is then read by the CCD detector. In reconstructive spectroscopy (right), an array of optical elements uniquely encodes the incoming light, and the resulting transmission is read on a CCD detector array. The known spectral properties of the resonator array are then used to computationally reconstruct the spectrum of the incident light.

The difference between conventional grating-based spectrometry^13^, conventional filter-array based spectrometry^9^,^14^, and reconstructive spectroscopy^4^,^7^,^10^,^15^,^16^ is illustrated in Fig. 1^ ^4. In conventional spectrometry, the incoming light is separated into its component wavelengths using diffractive or dispersive optics, and then read out by an array of light-detecting sensors. Although there are designs for compact diffraction spectrometers, the fundamental need to propagate the light after the diffraction grating makes reducing the footprint a substantial challenge. Filter-array spectrometers solve this issue by creating a planar 2D array of wavelength-sensitive bandpass filters. These can be placed directly over the camera detector, which leads to a more compact design. In both cases, they perform a one-to-one mapping of wavelengths to individual sensors, so that the recorded spectrum can be read out directly on the detector.

In contrast, spectrometry using algorithmic reconstruction relies on applying a sensing pattern to the signal, and then computationally reconstructing the spectrum using a best-fit algorithm. One way this can be experimentally achieved is by applying an array of spectral filters in front of a light detector^10^,^15^,^16^. Mathematically, with an incident spectrum *S*(λ) and *m* optical elements with known unique transmission spectra *T*_*i*_(λ), the detected signal *I*_i_ is described as equation (1):


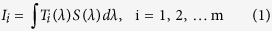


It is then possible to recover the original spectrum by applying a minimization algorithm to a best guess of *S* that fits the observed measurements *I*_*i*_. In the case of compressive sensing^17^,^18^,^19^, minimization of the L1 norm converges to a sparse solution^20^,^21^, and so using a compressive sensing reconstruction algorithm is well-suited to signals that can be efficiently represented in a relatively small number of measurements (or ‘sparse’) in some basis, a condition that is true for most natural signals of interest, including spectrometry^22^,^23^. In addition, this disassociation of the number of measurements from the number of data points in the recovered signal allows for a great deal of flexibility in the sensing pattern, as well as sensitivity and data compressibility. In our spectrometry method, we achieve this sampling by placing an array of optical resonators in front of a CCD detector, which uniquely encodes the light transmitted through each resonator, and a separate intensity value can therefore be recorded for each resonator. Although the properties of this type of spectrometer has been previously explored in theory^16^, where it is referred to as ‘Multiple-order staircase etalon spectrometry’, an experimental demonstration of its capabilities has not previously been achieved.

**Figure 2:**
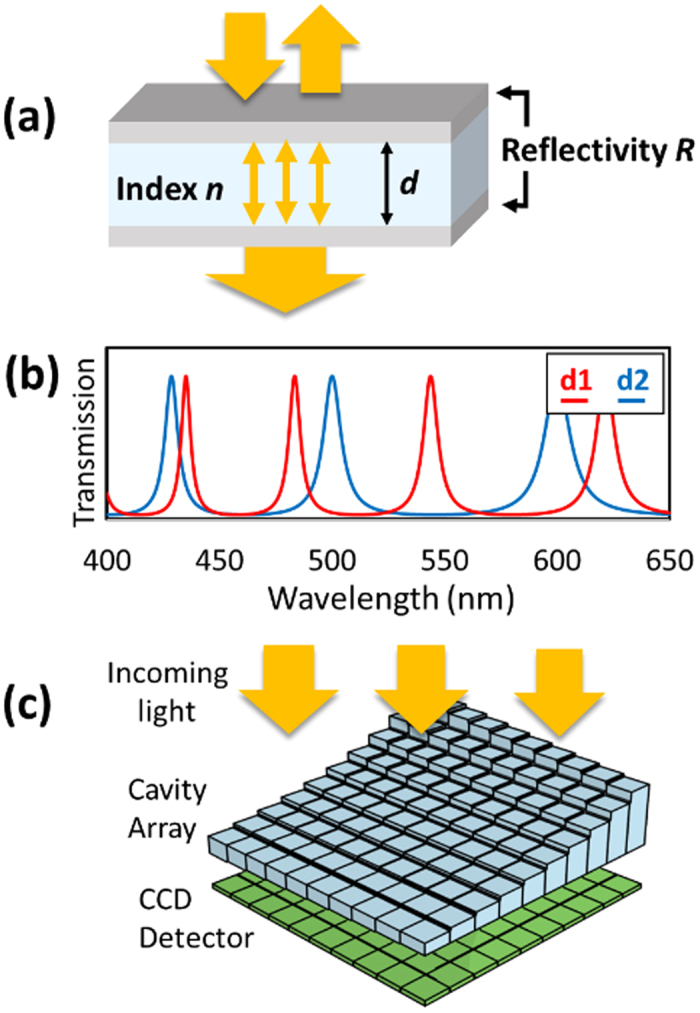
(**a**) An etalon consists of two semi-reflecting surfaces with reflectivity R separated by an optically transparent medium of index n and thickness d. Light reflecting between the two surfaces interferes with itself, creating a characteristic transmission pattern described by equation (1). (**b**) Two etalons of different thicknesses (d1 and d2) will have uniquely encoded transmission patterns. (**c**) A CCD detector positioned under an etalon array with unique thicknesses will record the encoded light after it is transmitted through the etalons. These measurements will be used to reconstruct the spectrum of the incident light.

The optical resonator we use for our spectral encoding is the etalon, also known as a Fabry-Perot cavity. It consists of a parallel pair of semi-reflective surfaces with reflectivity *R* separated by a distance *d* with an optically transparent medium of index *n* (see Fig. 2). When light of wavelength *λ* is incident at a normal angle on the etalon, the transmission *T* is given by equation (2):


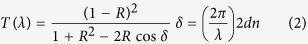


The resulting transmission pattern is a characteristic series of peaks in transmission where the reflections from the surfaces add constructively. Although varying the reflectance *R* of the etalon surfaces will change the finesse of the cavity, the position of the transmission peaks depends only on the optical thickness *dn* (Fig. 2b). As a result, varying the optical thickness in an etalon results in a tunable spectral encoding device for the transmitted light, with fine control over the exact transmission pattern. This high sensitivity to optical depth is instrumental for applications as diverse as laser cavities^24^, gravitational wave detectors^25^, and, of course, spectrometry^26^.

## Experiment

Our spectrometry method of etalon-array reconstructive spectrometry (EARS) is illustrated in Fig. 2c. We design a 2D etalon array such that each cavity has a unique optical thickness *d*, then image light transmitted through it with a CCD detector. This way, each individual etalon in the array acts as a unique spectral encoder, which can be quantitatively imaged by the CCD detector. Since the transmission signatures of the etalons are known, we can then reconstruct the original spectrum from the recorded 2D image.

**Figure 3:**
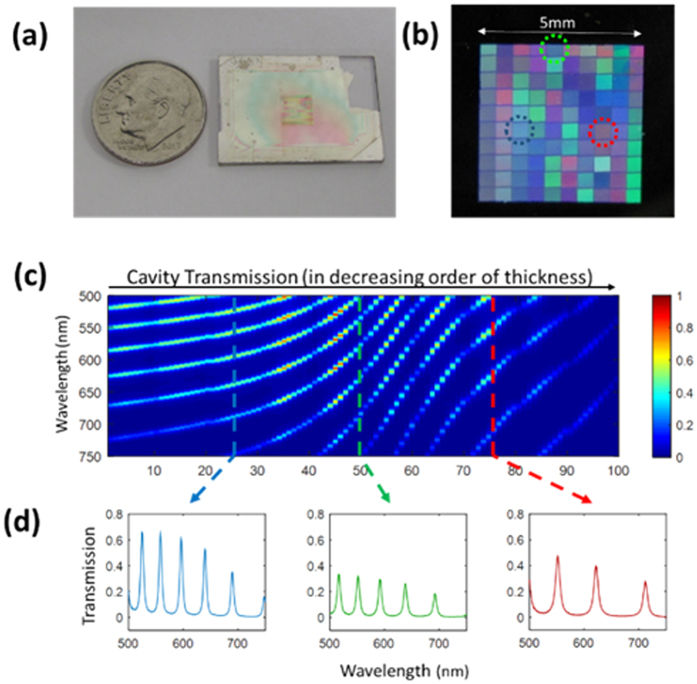
(**a**) A photo of our fabricated 10 × 10 etalon array next to a dime for scale. (**b**) A color photograph of the etalon array back-illuminated by room fluorescent lighting, showing cavity-dependent color transmission. Three specific etalons are circled with colored dotted lines. (**c**) The measured transmission spectra for all 100 cavities in order from thickest to thinnest. The three etalons circled in b are marked with dashed lines. (**d**) Transmission spectra for the three selected cavities.

A photograph of our prototype etalon array is shown in Fig. 3a,b. The semi-reflecting surfaces are 30 nm silver films (*R* ~ 0.7), and the optical spacing layer is a 700 nm SiO_2_ layer under a 10 × 10 PMMA step-structure with thicknesses from 0.8–2.8 microns, for a total cavity thickness variation from 1.5–3.5 microns in 100 steps (fabrication details available in Supplement 1). The cavity is then imaged with a CCD camera (Andor iXonEM 897). In order to characterize the etalon array, we overlaid an aperture over each individual cavity, and then measured the spectrum of the transmitted light (Fig. 3b,c) using a conventional grating-based spectrometer (Andor Shamrock SR-303i, measured from 400–970 nm at a resolution of 0.55 nm). In this way, the spectral transmission (*T*_*i*_) for each cavity was recorded to a high degree of accuracy.

To make our EARS measurement, we set our tunable bandpass filter to a specific bandwidth and center wavelength, and then we take an image of the CCD detector of the etalon array back-illuminated by the supercontinuum laser. We then digitally process the CCD image to extract an average intensity value for each etalon in the array. Finally, we input the known etalon response and the measured etalon brightness into a compressive sensing reconstruction algorithm (see Supplement 1), and reconstruct the spectrum of the incident light.

## Results

**Figure 4:**
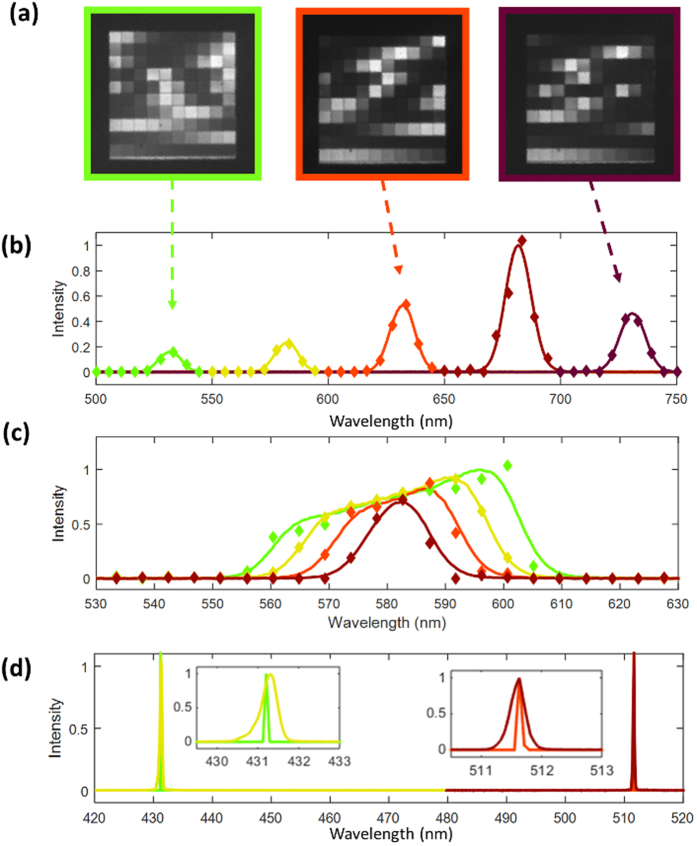
(**a**) Three CCD images of the etalon array illuminated by different light sources. (**b,c**) Reconstructed results (diamonds) compared with conventionally-measured spectra (solid lines) for several different incident spectra (marked with different colors). (**b**) Demonstrates the reconstructed spectra for a broadband source projected through several different band-pass filters at different peak wavelengths (530, 580, 630, 670, and 730 nm). (**c**) Shows the same source through band-pass filters at different nominal bandwidths (10, 20, 30, and 40 nm). (**d**) Recovery of laser spectra. Results from a conventional spectrometer show peaks at 431.32 and 511.6319 nm (Yellow, red), while our recovered spectra show peaks at 431.20 and 511.6327 nm (Green, orange).

The experimental performance of EARS is shown in Fig. 4. In our experiment, we were able to successfully recover a series of different spectra within a bandwidth of 400–750 nm (4b,c), limited by the bandwidth of our light source, though in practice the full bandwidth of our spectrometer is limited by the responsivity of our detector (for the Andor iXonEM 897, roughly 400–900 nm at a 50% quantum efficiency threshold). Slight differences between the conventionally measured and reconstructed spectra may be attributed to measurement errors in the system, although this error was greatly reduced with a calibration step using a known light source (see Supplement 1).

The spectral resolution of EARS, unlike traditional filter array spectrometers, is not directly tied to the number of detected measurements^15^. Instead, the achievable resolution is determined by the thickness range and finesse of the etalons^16^. In practice, we are able to achieve good spectral reconstruction at a sampling period of 4 nm, which translates into a Nyquist-limited resolution of 8 nm (See Supplement 1 for more detail).

However, the nature of compressive sensing allows it to be used very aggressively if the signal is known to be sparse in a particular basis. For instance, lasers are nearly monochromatic in wavelength, and so despite the resolution limit of our system, we can recover the peak wavelength of the laser to a very high degree of accuracy. In our experiment (Fig. 4d), we were able to recover the wavelength of two lasers to within 0.12 nm of the peak wavelength measured by a conventional spectrometer, far below the resolution limit. The accuracy of our measurement in this case is set by the accuracy of our calibration and the signal-to-noise ratio of the measured image. For future devices, an array with larger optical thicknesses can be fabricated in order to achieve even higher wavelength resolutions.

In addition, the total number of etalons in our cavity array will also set a limit on the spectra that can be successfully reconstructed. In particular, this limit is related to the *sparsity* of the measured spectrum, or in the number of components that are at or near zero. For instance, the spectrum of a laser is extremely sparse in wavelength, being nearly monochromatic, and so is well-suited for compressive sensing. On the other hand, a spectrum that looks like white noise would be the worst possible candidate for compressive sensing, as this signal would not be sparse in any basis. However, in the overwhelming majority of cases, signals of interest will not resemble random noise, which means that compressive sensing will usually be suitable^17^. The exact relationship between the sparsity of the signal and the measurement requirements is complex, but for a given signal there is a threshold number of measurements needed in order to reconstruct the signal correctly, and a larger number of etalons would allow for the reconstruction of spectra that are less sparse. In summary, the performance of EARS can be further improved in both resolution and flexibility by increasing the total thickness variation of the spectrometer and by increasing the total number of cavities in the array.

## Discussion

One particular advantage of EARS over other filter-array spectrometers is in its tradeoff of resolution with signal level. All spectrometers, to some extent, must sacrifice signal for resolution. In a conventional grating-based spectrometer, diffracted light must be divided among the CCD pixels for measurement, and so the signal read by a single pixel will scale as 1/N, where N is the total number of measurements made. A filter-array spectrometer, however, uses subtractive filter arrays instead of diffractive elements, and so in addition to spreading the light across all of the measurement pixels, each pixel must also block out all light except for the wavelength of interest. As a result, the signal received by each detector scales as 1/N^2^, which can result in low signal levels for high-resolution spectrometry. For EARS, however, the amount of light transmitted through an etalon is independent of the desired spectral resolution, and so preserves the 1/N signal scaling of conventional spectrometers. For example, we have demonstrated a sampling period of 5 nm across a 250 nm wavelength range, which has 51 separate data points. In a conventional bandpass-filter based spectrometer, each individual filter would admit only 1/51 of the incoming light, leaving each detector to read less than 2 percent of the incoming light. In contrast, averaged across all wavelengths, all of our cavities will transmit roughly 9 percent of the incident light (after passing through 2 layers of R = 0.7). This means that our etalon array spectrometer will have a speed and signal-to-noise advantage over comparable filter array spectrometers, and this relative advantage only increases as we increase the spectral resolution and number of array elements. Furthermore, if additional information is known about the type of spectra likely to be measured (e.g. if the spectra is known to be narrowband or broadband), then the parameters of the etalon can also be tuned to maximize the SNR of the measured light^16^.

In conclusion, we have experimentally demonstrated a spectrometer based on a 2D array of etalons on a CCD detector. This type of design allows for a compact, robust, and potentially low-cost design, and can be easily fabricated on the surface of the CCD detector itself for a very small footprint. Alternatively, with the widespread use of cameras embedded into personal cell phones, EARS is suitable as an add-on to an already existing camera, with the cell phone providing the computational power needed to run the reconstruction. Compared with other filter-based spectrometers, it possesses the advantage of a fixed average transmission value independent of the resolution of the spectrometer, which allows for a higher signal sensitivity. It is particularly well suited for tasks such as the accurate measurement of laser spectra due to the inherent sparsity of the signal. The resolution can be improved by fabricating the array with thicker optical cavities, and the bandwidth is limited only by the working range of the CCD detector. We anticipate this type of instrument to be useful for applications where small size, low cost, and simplicity are paramount, such as for lab-on-chip measurements, field spectrometry, embedded systems, and space applications.

## Additional Information

**How to cite this article:** Huang, E. *et al*. Etalon Array Reconstructive Spectrometry. *Sci. Rep.* **7**, 40693; doi: 10.1038/srep40693 (2017).

**Publisher's note:** Springer Nature remains neutral with regard to jurisdictional claims in published maps and institutional affiliations.

## Supplementary Material

Supplementary Materials
