# Supplementary material for: Etalon Array Reconstructive Spectrometry
Source: Sci Rep. 2017 Jan 11;7:40693. doi: 10.1038/srep40693 (PMC5225422; doi:10.1038/srep40693)
Supplement: Supplementary Materials [file srep40693-s1.pdf]

Etalon Array Reconstructive Spectrometry

Eric Huang, Qian Ma, Zhaowei Liu*

*zhaowei@ucsd.edu

Etalon Array Fabrication.

The etalon array was fabricated using standard clean-room techniques. First, a 30 nm silver layer was deposited on a glass substrate using sputtering deposition (Denton Discover 18). Then, a 700 nm layer of SiO2 was deposited using plasma-enhanced chemical vapor deposition (Oxford Plasmalab PECVD). Afterwards, a 2.8 micron thick layer of PMMA was spin-coated on top, and a thin 2.5 conductive gold layer was sputtered on the PMMA. Then, the sample was put in an electron beam lithography machine (Raith GmbH Raith50), and a 10x10 grid of squares with dimensions of 500 by 500 microns were exposed to a varying electron beam dose from 1.4 μC/cm2 to 80 μC/cm2 at an energy of 10 keV. The gold was then removed with a potassium iodide (KI) etchant, then the PMMA was developed in a solution of methyl isobutyl ketone (MIBK) solution for 5 minutes, followed by a 30 second rinse with isopropyl alcohol. This resulted in a 10 by 10 array of square pits in the PMMA with depths varying from 20 nm to 2 microns, for a total optical thickness range of roughly 1.5 to 3.5 microns including the SiO2 layer. After drying the sample under N2 air, we then added the final 30 nm silver layer using sputter deposition, as well as a thin 30nm protective layer of SiO2 on top to prevent oxidation of the silver.

Experimental Setup.

Our experimental setup is described in Fig. S1. Two light sources were used: an NKT SuperK EXB-6 supercontinuum laser, and a mercury lamp. The sources were coupled through a 100m multimode fiber to remove spatial coherence from the beam, and the supercontinuum laser was limited to a bandwidth of 500-750 nm with a tunable bandpass filter (NKT SuperK VARIA). The light was then roughly collimated from the fiber output with a reflective parabolic fiber coupler (Thorlabs RC12FC-P01). The light was then projected through the etalon array at a normal angle, and then through a pair of confocal 5x microscope objectives. This allowed us to insert an aperture into a conjugate image plane to accurately measure the transmission properties of each individual etalon. Then, the light passes through a beam splitter. On one half, the image is focused onto our CCD camera (Andor iXonEm 897), while the other half is sent to a conventional grating spectrometer (Andor Shamrock SR-303i with Andor iDus 420 CCD).

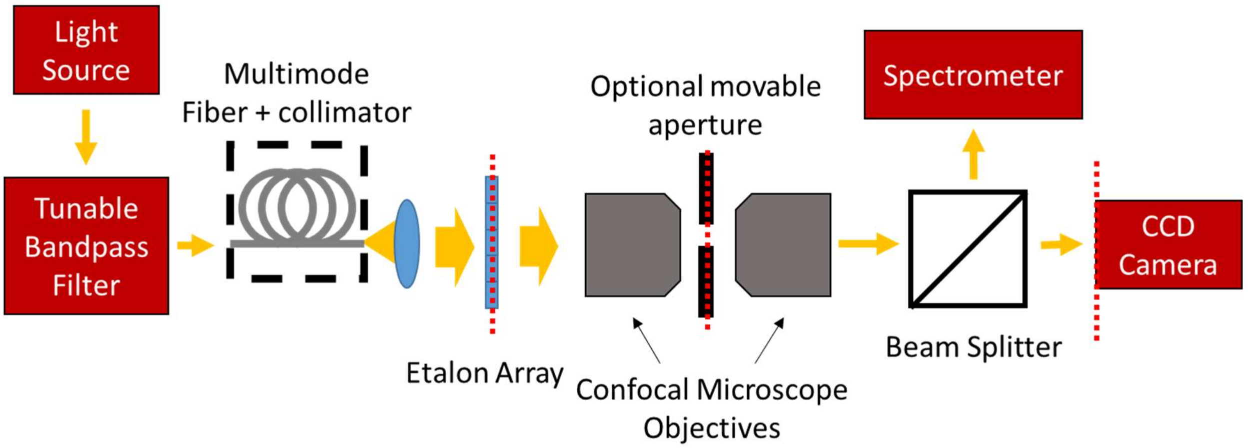

Figure 1: Our laser is sent through a tunable bandpass filter, then a multimode fiber to eliminate spatial coherence, then projected through the etalon array. The image of the array is focused onto an intermediate image plane with a microscope, where an aperture may be placed to measure the properties of the etalons individually. A second objective is used to focus the image of the etalon array, where a beam splitter diverts the light onto both a CCD camera and a spectrometer. Conjugate image planes to the etalon array are marked with dashed red lines.

Calibration and Error Correction.

Like all spectroscopy methods, some calibration may be necessary to ensure accurate results for EARS. For our method, the biggest issue is that although the transmission properties of the etalons are well characterized, our method for collimating the light led to a non-uniform illumination over the etalon array. Fortunately, this can be easily corrected by using a known spectrum as a calibration tool, and comparing the measured intensity of the detector compared with the expected intensity given the known spectrum for both the light and the etalon transmission. In Fig. S2, we show the quotient of the experimental and predicted measurements for illumination by four different known reference wavelengths, and can see that deviations from the expected value are similar for all 4 measurements. Therefore, calibration for our spectrometer can be achieved simply by taking the average deviation of all 4 calibration measurements, and dividing the experimentally measured value by this value for each individual cavity. These correction factors are then used for the remainder of our experiments.

$$Deviation = \frac{Expected\ Measurement}{Experimental\ Measurement}$$

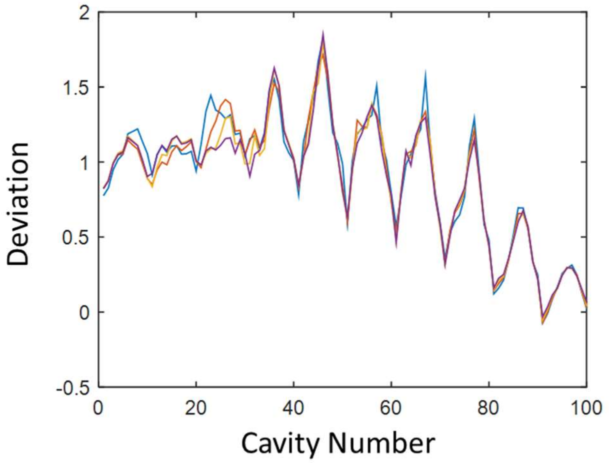

Figure 2: Since the incident spectrum is known, we can calculate what signal we expect to receive, and compare it with the signal we detect from each cavity. These results show that many etalons are consistently measured to be brighter or darker than their calibrated data would suggest. Since all of our measurements show similar deviations from the expected value, we can average them together and use this as a correcting factor for our reconstruction

Sensitivity to Angle Variation

The phase difference term for each round trip in a Fabry-Perot cavity is $\delta = \left(\frac{2\pi}{\lambda}\right) 2nl\cos\theta$, where λ is the wavelength, n is the index of refraction, *l* is the cavity length, and θ is the incident angle measured from normal. From this expression, it is easy to see that a FP cavity at a slight angle ($\cos\theta<1$) will behave identically to a FP cavity with a shorter optical length (*l*). The effect of this is that a deviation in angle will result in a uniform shift of the FP cavity response across the entire array. Therefore, as long as the angle remains fixed once the array has been measured and calibrated, the angle will not affect the signal to noise ratio of the measurement.

In the case of a non-uniformity of the angle across the etalon array, we can also estimate the minimum angular deviation needed to approach the change due to the thickness modulation. In our fabricated array, the stepwise variation for each etalon was 20 nm, while the maximum thickness was around 3500 nm, for a variation of 20/3500, or roughly .6%. In order to reach a .6% variation from a change in angle, the angle deviation from one cavity to the other would have to be $\cos^{-1}(1-20/3500)$, or roughly 6 degrees. Since the total angular spread from our collimated beam is well below that (<1 degree), we do not need to worry about sampling degeneracies due to angular spread.

Compressive Sensing Algorithm.

The algorithm used for the compressive sensing reconstruction is described by Kim et. al [1], which uses the L1-norm as a basis for minimization. The basis for the reconstruction was in the wavelength basis. For the spectroscopic reconstruction, the basis vectors of the sensing matrix were the individually measured spectral transmissions of the FP cavities. No additional constraints

(non-negativity, etc) was imposed on the reconstruction. The reconstruction code was executed using a conventional laptop computer running commercial MATLAB software. Typical reconstruction times were less than 30 seconds.

Reconstruction Limits

The conditions necessary for successful compressive sensing reconstruction in our method can be described as: [2]

$$\boldsymbol{m \geq C \cdot S \cdot \mu^2 log\, n}$$

where m is the number of etalons, S is the sparsity of the signal, μ is the coherence between the sensing basis and the signal basis, n is the total number of elements in the reconstructed spectrum, and C is some constant. It is not possible to know beforehand what the exact requirements are, but in general a larger number of etalons (m) will allow us to recover larger spectra (n).

Resolution Limit.

The resolution limit of a spectrometer based on multiple order etalon transmission has been previous explored in other work [3], and predicts a wavenumber Nyquist-limited resolution:

$$\Delta\sigma > \frac{1}{2d_r F}$$

where d_r is the range of cavity lengths and $F = \pi\sqrt{R}/(1-R)$ is the finesse of the cavity, with R as the reflectivity of the etalon surfaces. Consequently, this means that the resolution in wavelength space varies depending on the wavelength, but if we use our experimental values (d_r=2000 nm and R=.7), we can expect a Nyquist resolution close to 10 nm at a wavelength of 600nm.

To test this, we simulated measurements on our etalon array for a Gaussian spectrum with a peak centered at 600 nm at a variety of sampling periods in wavelength (Supp. Fig. 3b). While the simulation is successful for sampling periods down to 4 nm, as we decrease the sampling period, the algorithm begins to converge towards solutions that are no longer representative of the waveform. Since this sampling period is half of the Nyquist resolution, we can say that our simulated resolution limit is around 8 nm, a figure that is reasonably close to the predicted value.

Qualitatively, this value is reasonable. The full-width half maximum (FWHM) of our thickest cavity was about 7 nm (Supp. Fig. 3a), and since we can consider these peaks to be the point-spread function of our sampling pattern, we expect to be able to resolve features at a comparable level of detail.

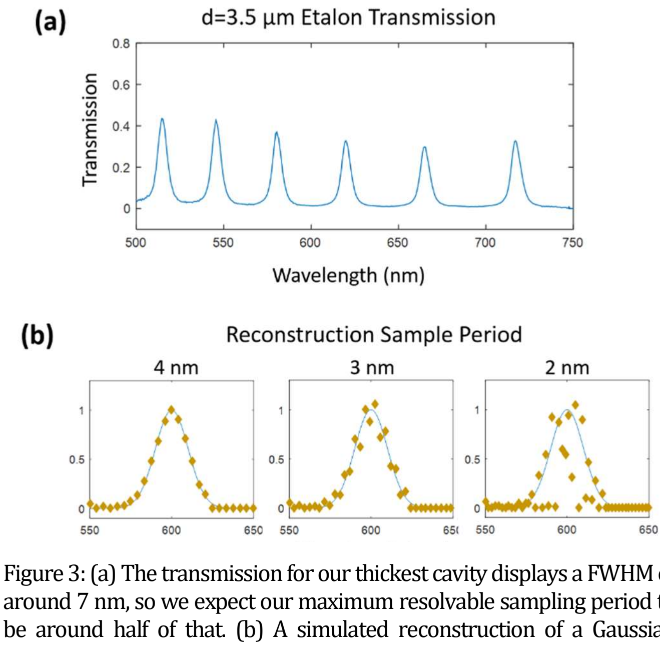

Figure 3: (a) The transmission for our thickest cavity displays a FWHM of around 7 nm, so we expect our maximum resolvable sampling period to be around half of that. (b) A simulated reconstruction of a Gaussian spectrum at multiple sampling periods (4, 3, and 2 nm).

References

1. S. B. Kwangmoo Koh, Seung-Jean Kim, "An interior-point method for large-scale l1-regularized logistic regression," J. Mach. Learn. Res. **8**, 1519–1555 (2007).
2. E. J. Candes and M. B. Wakin, "An Introduction To Compressive Sampling," IEEE Signal Process. Mag. **25**, 21–30 (2008).
3. O. A. Sw, M. K. Yetzbacher, C. W. Miller, a. J. Boudreau, M. Christophersen, and M. J. Deprenger, "Multiple-order staircase etalon spectroscopy," **9101**, 910104 (2014).
